# Effect of goal-directed haemodynamic therapy guided by non-invasive monitoring on perioperative complications in elderly hip fracture patients within an enhanced recovery pathway

**DOI:** 10.1186/s13741-022-00277-w

**Published:** 2022-08-10

**Authors:** Juan V. Lorente, Francesca Reguant, Anna Arnau, Marcelo Borderas, Juan C. Prieto, Jordi Torrallardona, Laura Carrasco, Patricia Solano, Isabel Pérez, Carla Farré, Ignacio Jiménez, Javier Ripollés-Melchor, Manuel I. Monge, Joan Bosch

**Affiliations:** 1 Anaesthesia and Critical Care Department, Juan Ramón Jiménez Hospital, Ronda Norte s/n, 21590 Huelva, Spain; 2 School of Medicine and Health Sciences, International University of Catalonia (UIC), Barcelona, Spain; 3 Fluid Therapy and Hemodynamic Group of the Hemostasis, Transfusion Medicine and Fluid Therapy Section, Spanish Society of Anesthesia and Critical Care (SEDAR), Madrid, Spain; 4 Department of Anaesthesiology, Althaia Xarxa Assistencial Universitària, Manresa, Spain; 5 Central Catalonia Chronicity Research Group (C3RG), Research and Innovation Unit, Althaia Xarxa Assistencial Universitària, Manresa, Spain; 6 Centre d’Estudis Sanitaris i Socials, (CESS), Universitat de Vic–Universitat Central de Catalunya (UVIC-UCC), Vic, Spain; 7 Clinical Management Anesthesiology Unit, Resuscitation and Pain Therapy, Virgen del Rocio Hospital, Sevilla, Spain; 8 Anesthesia and Critical Care Department, Infanta Leonor Hospital, Madrid, Spain; 9 Intensive Care Unit, Hospital Universitario SAS, Jerez de la Frontera, Spain

**Keywords:** Enhanced recovery after surgery, Enhanced recovery pathway, Fluid therapy, Goal-directed haemodynamic therapy, Hip fracture, Intraoperative complications, Mortality, postoperative complications

## Abstract

**Background:**

Goal-directed haemodynamic therapy (GDHT) has been shown to reduce morbidity and mortality in high-risk surgical patients. However, there is little evidence of its efficacy in patients undergoing hip fracture surgery. This study aims to evaluate the effect of GDHT guided by non-invasive haemodynamic monitoring on perioperative complications in patients undergoing hip fracture surgery.

**Methods:**

Patients > 64 years undergoing hip fracture surgery within an enhanced recovery pathway (ERP) were enrolled in this single-centre, non-randomized, intervention study with a historical control group and 12-month follow-up. Exclusion criteria were patients with pathological fractures, traffic-related fractures and refractures. Control group (CG) patients received standard care treatment. Intervention group (IG) patients received a GDHT protocol based on achieving an optimal stroke volume, in addition to a systolic blood pressure > 90 mmHg and an individualized cardiac index. No changes were made between groups in the ERP during the study period. Primary outcome was percentage of patients who developed intraoperative haemodynamic instability. Secondary outcomes were intraoperative arrhythmias, postoperative complications (cardiovascular, respiratory, infectious and renal complications), administered fluids, vasopressor requirements, perioperative transfusion, length of hospital stay, readmission and 1-year survival.

**Results:**

In total, 551 patients (CG=272; IG=279) were included. Intraoperative haemodynamic instability was lower in the IG (37.5% vs 28.0%; p=0.017). GDHT patients had fewer postoperative cardiovascular (18.8% vs 7.2%; *p* < 0.001), respiratory (15.1% vs 3.6%; *p*<0.001) and infectious complications (21% vs 3.9%; *p*<0.001) but not renal (12.1% vs 33.7%; *p*<0.001). IG patients had less vasopressor requirements (25.5% vs 39.7%; *p*<0.001) and received less fluids [2.600 ml (IQR 1700 to 2700) vs 850 ml (IQR 750 to 1050); *p*=0.001] than control group. Fewer patients required transfusion in GDHT group (73.5% vs 44.4%; *p*<0.001). For IG patients, median length of hospital stay was shorter [11 days (IQR 8 to 16) vs 8 days; (IQR 6 to 11) *p* < 0.001] and 1-year survival higher [73.4% (95%CI 67.7 to 78.3 vs 83.8% (95%CI 78.8 to 87.7) *p*<0.003].

**Conclusions:**

The use of GDHT decreases intraoperative complications and postoperative cardiovascular, respiratory and infectious but not postoperative renal complications. This strategy was associated with a shorter hospital stay and increased 1-year survival.

**Trial registration:**

ClinicalTrials.gov NCT02479321.

**Supplementary Information:**

The online version contains supplementary material available at 10.1186/s13741-022-00277-w.

## Background

Hip fracture represents an increasingly serious public health problem with a significant impact on life expectancy and economic burden (Veronese and Maggi 2018). Patients with hip fractures are at high risk of perioperative complications (Reguant et al. 2019) due to a limited cardiorespiratory reserve when facing the fracture and surgery-associated stress (Cowan et al. 2017). Moreover, postoperative complications related to hip fracture are a known independent risk factor for mortality (Griffiths et al. 2021).

Enhanced recovery pathways (ERP) comprise perioperative evidence-based care interventions designed to improve outcomes after surgery (Ljungqvist et al. 2017). Perioperative haemodynamic optimization is a key element of the ERP (Miller et al. 2015). The goal-directed haemodynamic therapy (GDHT) has been shown to reduce morbidity and mortality in high-risk surgical patients (Giglio et al. 2016). Nevertheless, in daily clinical practice, patients with hip fracture are usually intraoperatively monitored with routine haemodynamic parameters such as blood pressure and heart rate (Reguant et al. 2019). However, these standard physiological variables result insufficient for assessing an adequate balance between oxygen delivery (DO_2_) and consumption (VO_2_) (Lugo et al. 1993). This DO_2_/VO_2_ imbalance may eventually lead to intraoperative tissue hypoperfusion, facilitating the appearance of postoperative complications (Merry and Mitchell 2018).

Currently, there are monitoring platforms that provide advanced haemodynamic parameters to guide GDHT in a non-invasive way to avoid the complications of invasive techniques (Teboul et al. 2016).

The present study aimed to evaluate the effect of GDHT guided by non-invasive haemodynamic monitoring on perioperative complications in patients undergoing surgical hip fracture repair within an ERP.

## Methods

This manuscript was written according to CONSORT statement. The study was approved by an independent Ethics Committee (Fundació Unio Catalana Hospitals) on 27 January 2015 (CEIC 15/03). All patients signed an informed consent to participate. The study was conducted according to the Declaration of Helsinki and all local legal and regulatory requirements. Trial registration: NCT02479321 (24/06/2015).

### Study design

This is a single-centre, non-randomized, hospital-based intervention study with a historical control group (CG) and 12-month follow-up after hospital discharge.

### Inclusion/exclusion criteria

Patients over 64 years with hip fracture within an ERP who underwent surgical treatment were included.

Exclusion criteria were as follows: patients with pathological fractures, traffic-related fractures and refractures; patients with known contraindication or limitations to advanced haemodynamic monitoring with ClearSight® system and EV1000 platform (Edwards Lifesciences, Irvine, USA) (Saugel et al. 2015); patients with Raynaud disease, with aortic valve prosthesis, proximal aortic aneurysm, known intra-cardiac shunts; moderate to severe mitral or aortic regurgitation; moderate to severe aortic or mitral stenosis; patients with poor-quality arterial waveform signal (see below) and patients with significant preoperative psychomotor agitation.

### Conduct of the study

#### Perioperative management common to both groups

Both groups were treated during the perioperative period in a multidisciplinary ERP unit created in 2010 exclusively dedicated to patients undergoing hip fracture repair (Reguant et al. 2019).

This unit’s objectives were to optimize patient health status before surgery, minimize preoperative stress, prevent and/or treat electrolyte imbalance, prevent and/or treat cardiovascular, respiratory, infectious and cognitive disorders, improve nutritional status and reduce surgical delay. The team comprised orthopaedic surgeons, anaesthesiologists, internists, a nurse case manager, a social worker, a physiotherapist and a nutritionist.

The main interventions of this multidisciplinary ERP unit for patients with hip fracture are shown in Table 1.

**Table 1:** Main interventions of enhanced recovery pathway unit for hip fracture patients

Preoperative period	Intraoperative period	Postoperative period
-Specialized hip fracture ward - Internist support - Assessment by anaesthesiologist - Nursing aids - Intravenous fluids - Monitor oxygen saturation/8 h. Oxygen therapy when < 92% and maintenance until 48 h after surgery - Pain control: avoiding opioids if possible - Carbohydrate loading until 2 h before surgery. - Protocol for patients who received antiplatelet drugs or oral anticoagulants on admission.^a^ - Prioritize surgery within 48 h on admission in patients with medical stable condition.	- Prevention of intraoperative hypothermia - Intraoperative nausea and vomiting prophylaxis - Prophylactic antibiotic 30 min before surgical incision ^b^ - Avoid intrathecal opioids - Performance of peripheral nerve blocks	- Specialized hip fracture ward - Internist support - Nursing aids - Postoperative fluids should be stopped when possible, in favour of early oral intake. - Monitor oxygen saturation/8 h. Oxygen therapy when < 92% and maintenance until 48 h after surgery - Optimal postoperative analgesia, preferably with intraoperative peripheral nerve blocks and NSAIDs - Deep vein thrombosis prevention - Early respiratory physiotherapy - Early and standardized mobilization 24h after surgery. - Early urinary catheter removal
**Perioperative interventions**		
- Gastric ulcer prophylaxis iv/24 h. - To avoid using opioids and/or benzodiazepines. - Screening and treatment when appropriate of urinary infection - Bladder catheterisation only in case of incontinency or when needing to monitor renal and/or cardiac function. - Treatment protocol for anaemia when haemoglobin was < 13 g/dl on admission. Transfusion was administered if haemoglobin level was < 8 g/dl and to patients with cardiorespiratory disease and/or haemodynamic instability when haemoglobin level was < 10 g/dl.		

^a^ Surgery was postponed for 4 days in patients who, at admission, had been administered acetylsalicylic acid >100 mg, triflusal >300 mg or clopidrogel/ticlopidine. Surgery was postponed in patients who were on OAC treatment at admission, until INR < 1.5

^b^ 2 g cefazolin in intramedullary nail surgery in 100 ml saline, or cefuroxime and teicoplanin in prosthesis surgery (in a total of 200 ml saline)

##### Intraoperative period

All subjects received standard of care with a 3-lead electrocardiogram, pulse oximetry and two peripheral intravenous lines. Patients in both groups received standard measures to maintain oxygen saturation by pulse oximetry >94% and heart rate <100 beats/min. Anaesthetic technique was at the discretion of the anaesthetist.

##### Post-anaesthetic care unit (PACU)

After surgery, patients were treated in the PACU. The attendant physician determined discharge from this unit according to the local protocol.

#### Study arms

##### Control group

Data from patients who underwent surgery for hip fracture between October 2010 and November 2011 with follow-up to December 2012 were used for the CG (Reguant et al. 2019).

Haemodynamic management was at the discretion of the attending anaesthetist, using fluid therapy with crystalloids (0.9% saline, lactated Ringer or Isofundin®), colloids (Voluven®, Gelaspan ®) and/or cardiovascular drugs (in bolus—ephedrine, or continuous infusion—noradrenaline, dobutamine).

Non-invasive, intermittent arterial pressure measurement was obtained at least every 5 min using a cuff (Dahtex Ohmeda-GE S/5 Aespire ®).

##### Intervention group

Data from patients who underwent surgery for hip fracture between June 2015 and February 2018 with follow-up to March 2019 were used as the IG.

Pre- and intraoperative non-invasive haemodynamic monitoring was conducted using ClearSight® monitor (Edwards Lifesciences, Irvine, USA). This monitoring system is based on the volume clamp method to continuously measure arterial pressure and the Physiocal method that periodically recalibrates the system (Saugel et al. 2015). Baseline haemodynamic measurements were taken when the Physiocal value exceeded 30 (Wesseling et al. 1995). If a Physiocal value over 30 was not obtained after 7 min monitoring, the patient was excluded due to a poor-quality arterial waveform signal (Wesseling et al. 1995).

Haemodynamic optimisation was performed according to the following GDHT protocol.

**Fig. 1:**
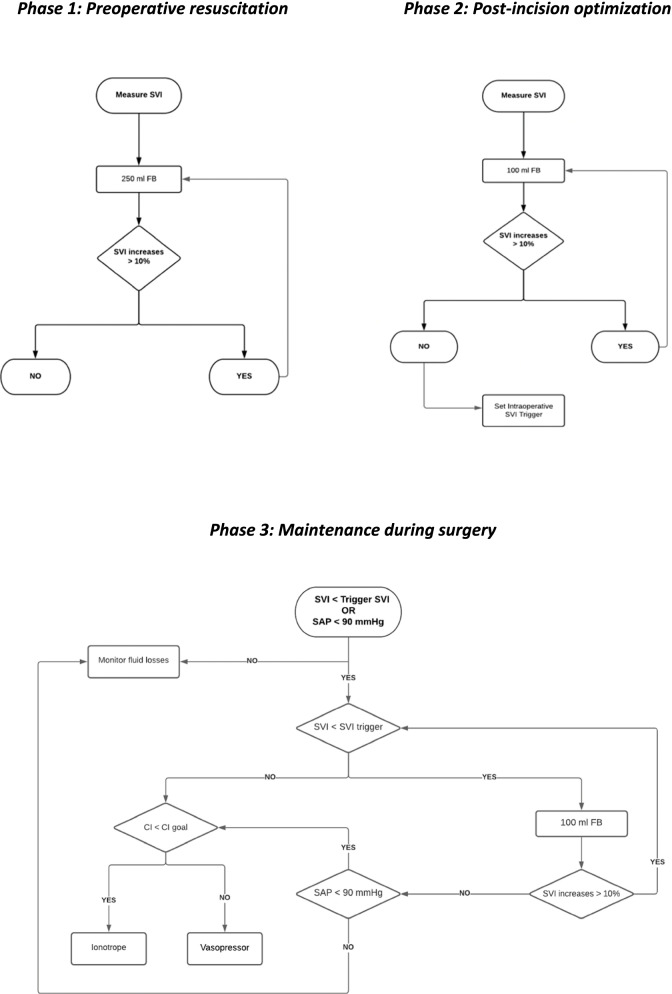
Algorithms for goal-directed haemodynamic therapy phases

GDHT protocol (Fig. 1)

Three groups of cardiac index (CI) goals were formed according to age and prior functional capacity expressed in metabolic equivalents (METS) (Montenij et al. 2014) Additional file 1.

Fluids were given based on a protocolized haemodynamic algorithm to achieve and maintain an adequate indexed stroke volume (SVI) using crystalloids (0.9% Saline, Lactated Ringer or Isofundin ®) or colloids (if preoperative glomerular filtration rate was above 60 mL/min using Modification of Diet in Renal Disease (MDRD) equation (Ishihara 2014)- Voluven ®, Gelaspan ®). Choice of fluid type was based on anaesthesiologist criteria.

If a fluid bolus (FB) was not indicated and/or the target perfusion pressure was not been achieved with its infusion, vasopressor was administered to maintain systolic arterial pressure (SAP) above 90 mmHg (in bolus—ephedrine, phenylephrine, or continuous infusion—noradrenaline) or continuous infusion of dobutamine was added to achieve in addition, the individualized CI goal.

Phase 1: Preoperative resuscitation

On arrival in the surgical area, patients received a FB of 250 ml of 5 min. If SVI increased by 10% or more (*First Fluid Bolus Responder*), the fluid bolus was repeated (Cecconi et al. 2011). Fluid boluses of 250 ml were repeated until the SVI failed to increase by 10%.

Once preoperative resuscitation was completed, prophylactic antibiotic was infused (Table 1). This fluid contribution covered the estimated insensible losses during surgery (Jacob et al. 2007).

Phase 2: Post-incision optimisation

Post-incision optimisation began 15 min after the surgical incision, if the haemodynamic stabilization was achieved (SAP and heart rate variation < 10% for 3 min); meanwhile, the haemodynamic priority was the maintenance of arterial pressure above goal set (Tassoudis et al. 2011).

Haemodynamic optimisation consisted of a 100-ml fluid bolus administered of less than 3 min (Guinot et al. 2015; Mallat et al. 2015; Marik 2015; Muller et al. 2011). If SVI rose >10%, the 100 ml fluid bolus was repeated. The trigger SVI during surgery was calculated by subtracting 10% from the SVI obtained from the last positive 100 ml fluid bolus (Muñoz et al. 2016).

Phase 3: Maintenance during surgery

If at least one of the following objectives, SVI>SVI trigger and /or SAP>90mmHg, were not achieved, SVI was analysed:

- If it was lower than the trigger SVI, a 100 ml FB was administered.
- If SVI was higher than trigger SVI, we look at the CI.
  - If its value was under goal level, dobutamine was added.
  - When CI was above goal level, a vasopressor was chosen.

After each therapy, we re-evaluated the achievement of SAP and SVI goals.

### Measurements and data handling

#### Procedure

Intraoperative haemodynamic parameters (arterial pressure, heart rate, SpO_2_ in CG and also CI and SVI in IG) were registered at 15-min intervals. Haemodynamic instability, between intervals, was registered as an event in the next record. Fluids and cardiovascular drugs used from the patient’s arrival in the surgical area to their admission to the PACU were collected. In both groups, the evaluation of intraoperative complications was based on the intraoperative anaesthesia charts, whereas the postoperative complications were documented in the clinical course and hospital discharge report.

Post-discharge follow-up consisted of a structured telephone interview at 1, 3, 6 and 12 months after surgery. When the information could not directly be obtained from the patients (including deceased patients), the interview was done with next of kin or carer.

#### Assessment of outcomes

##### Primary outcome measures

The primary outcome was the percentage of patients who developed intraoperative haemodynamic instability, defined as one measurement of SAP < 90 mmHg in the CG and for at least 1 min in the IG and/or the need for a bolus of vasoconstrictor.

##### Secondary outcome measures

- Intraoperative arrhythmias: defined as electrocardiographic evidence of cardiac rhythm disturbance.
- Postoperative complications, grouped as follows:
  - Major cardiovascular complications*: acute myocardial infarction, acute pulmonary oedema, ischemic stroke, pulmonary thromboembolism and cardiorespiratory arrest*.
  - Minor cardiovascular complications: *haemodynamic instability*, defined as one measurement of SAP < 90 and *arrhythmias*.
  - Respiratory*: hypoxia*, defined as oxygen saturation <92%. Other respiratory complications: *decompensation of chronic obstructive pulmonary diseas*e, *acute respiratory infection* (clinical and radiological diagnosis and antibiotic treatment) and others.
  - Renal: presence of at least one of the following: *oligoanuria*, defined as urine output under 0.5ml/kg per hour, including absence of urine output. *Acute renal failure*, defined as an increase in urea > 50 mg/dl and creatinine levels > 1.09 mg/dl in any analysis during admission.
  - Infections: *surgical wound (*infection within 30 days after surgery that involves only skin and subcutaneous tissue of the incision), *urinary* (positive urine culture causing patient’s symptoms and which were not present on admission to hospital), *systemic* (fever >38 °C and positive blood antigen test with appropriate antimicrobial therapy instituted by a physician)*.*
  - Surgical reintervention during hospital stay.
- Total intraoperative volume and type of administered fluids, doses of cardiovascular drugs used, perioperative packed red blood cell transfusion, length of hospital stay, readmission within 30 days of surgery, and survival within 12 months after surgery.

### Statistical analysis

#### Sample size

The rate of intraoperative haemodynamic instability described with standard of care was 37.5% (Reguant et al. 2019). We planned a relative risk reduction of 30% in IG.

To achieve a power of 80% using a bilateral *χ*^2^ test for two independent samples with a level of significance of 0.05, 538 patients had to be included (269 patients in each group). With a potential dropout of 5%, 568 patients were included.

The percentage of patients who developed one or more postoperative complications in CG was 45.2%. A meta-analysis by Grocott and colleagues suggested a RR reduction of 0.68 for complications in patients undergoing major surgery (Grocott et al. 2013). A sample size of 568 patients, 284 in each group, would have 80% power to detect a reduction of at least 22% in the number of IG patients presenting one or more postoperative complications, using a bilateral *χ*^2^ test for two independent samples.

#### Statistical analysis

Categorical variables were presented as absolute values and relative frequencies. Continuous variables are summarized as means and standard deviation for normal distribution and by the median and interquartile range (IQR) (25th to 75th percentiles) for non-normal distributions.

In the bivariate analysis, we used the Student’s *t*-test or the non-parametric Mann–Whitney *U* test for continuous variables. We used the *χ*^2^ test for categorical variables, and Fisher’s exact test or bilateral exact *p*-values in contingency tables when the expected frequencies were less than five.

One-year survival Kaplan–Meier curves were constructed, and the log-rank test was used to compare them. Crude and adjusted hazard ratios and confidence intervals (CI 95%) were calculated using Cox proportional regression models. The proportionality of hazards was verified by examining Schoenfeld residual plots.

Outcomes were analysed on an intention-to-treat basis. The level of statistical significance was two-sided 5% (*p* < 0.05). The IBM SPSS Statistics v.26 (IBM Corporation®, Armonk, New York) and Stata v.14 (StataCorp LP®, College Station, Texas) programmes were used for statistical analysis.

## Results

**Fig. 2:**
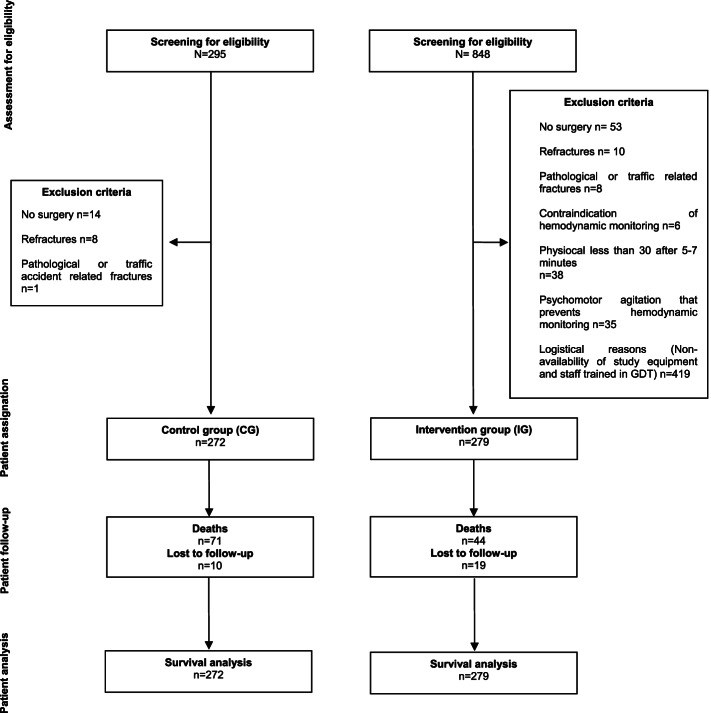
Flow chart of patients during recruitment and 12-month follow-up

A total of 551 patients were recruited. Study flowchart is shown in Fig. 2. Table 2 shows the baseline characteristics of the 272 patients in CG and the 279 patients in IG.

**Table 2:** Baseline characteristics according to group allocation

	Control (CG) ***n***=272	Intervention (IG) ***n***=279
**Age**	84.9 ± 6.2	85.2 ± 7.4
65 to < 75 years	15 (5.5%)	30 (10.8%)
75 to < 85 years	125 (46.0%)	97 (34.8%)
≥ 85 years	132 (48.5%)	152 (54.5%)
**Gender**		
Male	84 (30.9%)	68 (24.4%)
Female	188 (69.1%)	211 (75.6%)
**ASA**		
I–II	85 (31.2%)	41 (14.7%)
III–IV	187 (68.8%)	238 (85.3%)
**Charlson comorbidity index**		
Absence de comorbidity (0–1)	125 (46.0%)	109 (39.1%)
Low comorbidity (2)	50 (18.4%)	57 (20.4%)
High comorbidity (3 or more)	97 (35.7%)	113 (40.5%)
**Cardiovascular history**		
Valvulopathy	21 (7.7%)	31 (11.1%)
Arrhythmia	73 (26.8%)	58 (20.8%)
Ischemic cardiopathy	17 (6.3%)	29 (10.4%)
Pulmonary thromboembolism (PTE)	1 (0.4%)	3 (1.1%)
Hypertension	177 (65.1%)	212 (76.0%)
**Total number of drugs**	6 (IQR 4 to 8)	7 (IQR 5 to 10)
≤ 4 drugs	89 (32.7%)	68 (24.4%)
> 4 drugs	183 (67.3%)	211 (75.6%)
**Type of fracture**		
Intra-capsular	121 (44.5%)	124 (44.4%)
Extra-capsular	151 (55.5%)	155 (55.6%)
**Haemoglobin at admission**		
Haemoglobin > 12 g/dl	159 (58.5%)	169 (60.6%)
Haemoglobin ≤ 12 g/dl	113 (41.5%)	110 (39.4%)
**Creatinine at admission**		
Creatinine ≤ 1.09 mg/dl	178 (65.7%)	175 (62.7%)
Creatinine > 1.09 mg/dl	93 (34.3%)	104 (37.3%)
**Anaesthesia**		
General	28 (10.3%)	28 (10.0%)
Spinal	244 (89.7%)	251 (90.0%)
**Type of implant**		
Hip prosthesis	103 (37.9%)	102 (36.6%)
Dynamic hip screw	122 (44.9%)	57 (20.4%)
Intramedullary nail	43 (15.8%)	118 (42.3%)
Others	4 (1.5%)	2 (0.7%)
**Surgical delay**		
≤ 48 h	150 (55.1)	191 (68.5)
> 48 h	122 (44.9)	88 (31.5)
**Surgery time (minutes)**	80 (IQR 65 to 105)	90 (IQR 70 to 120)

Mean ± Standard deviation; *n* (%); median (IQR 25th percentile to 75th percentile)

Mean age was 84.9 years (69.1% female) in the CG and 85.2 years (75.6% female) in IG. Patients in the IG had a worse health status according to the criteria of ASA (III–IV 68.8% vs 85.3%; *p* < 0.001). A higher percentage of patients with intake of more than 4 drugs (67.4% vs 75.6%; *p*=0.03) was observed in the IG. No significant differences between groups were observed according to type of fracture; despite this, use of intramedullary nail increased in the IG (15.8% vs 42.3%; *p*<0.001). Surgical time was higher in the IG (80 min vs 90 min; *p*<0.004). There were no differences between anaesthesia techniques in two groups. Surgery was performed within 48 h of admission in 55.1% in the CG vs 68.5% of patients in the IG (*p*=0.001).

### Primary outcome

Details of intraoperative complications are shown in Table 3. The number of patients with intraoperative haemodynamic instability was lower in IG (37.5% vs 28.0%; *p*=0.017). The median number of episodes of intraoperative haemodynamic instability in IG was lower than in the CG [2 (IQR 1 to 4) vs 1 (IQR 1 to 2), *p*<0.001].

**Table 3:** Main and secondary outcomes at 1-year follow-up

	Control (CG) ***n***=272	Intervention (IG) ***n***=279	***p***-value
**Intraoperative complications**			
Hemodynamic instability	102 (37.5%)	78 (28.0%)	0.017^a^
No. of episodes of hemodynamic instability^e^	2 (IQR 1 to 4)	1 (IQR 1 to 2)	<0.001^b^
Arrhythmias	6 (2.2%)	2 (0.7%)	0.172^c^
**Postoperative complications**	123 (45.2%)	118 (42.3%)	0.489^a^
**Cardiovascular**	51 (18.8%)	20 (7.2%)	<0.001^a^
**Major**	12 (4.4%)	11 (3.9%)	0.783^a^
Myocardial infarction	1 (0.4%)	0 (0.0%)	0.494^c^
Cardiorespiratory arrest	3 (1.1%)	6 (2.2%)	0.505^c^
Acute pulmonary edema	8 (2.9%)	5 (1.8%)	0.374^a^
Pulmonary thromboembolism	0 (0.0%)	0 (0.0%)	-
Cardiorespiratory arrest	0 (0.0%)	0 (0.0%)	-
**Minor**	40 (14.7%)	12 (4.3%)	<0.001^a^
Haemodynamic instability	34 (12.5%)	5 (1.8%)	<0.001^a^
Arrhythmias	4 (1.5%)	7 (2.5%)	0.384^a^
Others	2 (0.7%)	1 (0.4%)	0.620^c^
**Respiratory**	41 (15.1%)	10 (3.6%)	<0.001^a^
Hypoxia	17 (6.3%)	2 (0.7%)	<0.001^a^
Decompensation of chronic obstructive pulmonary disease	6 (2.2%)	1 (0.4%)	0.066^c^
Acute respiratory infection	16 (5.9%)	5 (1.8%)	0.012^a^
Others	3 (1.1%)	2 (0.7%)	0.682^c^
**Renal**	33 (12.1%)	94 (33.7%)	<0.001^a^
**Infections**	57 (21.0%)	11 (3.9%)	<0.001^a^
Surgical wound	8 (2.9%)	0 (0.0%)	0.003^c^
Urinary	47 (17.3%)	10 (3.6%)	<0.001^a^
Systemic	3 (1.1%)	2 (0.7%)	0.682^a^
**Secondary to spinal anaesthesia**			
Hematoma/infection/neurological lesion	0 (0.0%)	0 (0.0%)	-
**Surgical reintervention**	6 (2.2%)	0 (0.0%)	0.014^c^
**Length of stay** (days)	11 (IQR 8 to 16)	8 (IQR 6 to 11)	<0.001^b^
**Destination after discharge**			0.327^a^
Convalescence	128 (49.8%)	142 (52.0%)	
Family home	81 (31.5%)	71 (26.0%)	
Residence	48 (18.7%)	60 (22.0%)	
**Thirty-day readmission**	29 (11.3%)	21 (7.7%)	0.157^a^
**Survival**			0.003^d^
1 month	91.2% (87.1 to 94.0%)	96.8% (93.9 to 98.3%)	
3 months	88.6% (84.2 to 91.8%)	95.3% (92.1 to 97.3%)	
6 months	83.0% (78.0 to 87.0%)	90.2% (86.0 to 93.2%)	
12 months	73.4% (67.7 to 78.3%)	83.8% (78.8 to 87.7%)	

Mean ± Standard deviation; *n* (%); median (range *x* to *y*) or median (IQR 25th percentile to 75th percentile)

^a^ Pearson *χ*^2^; ^b^ Mann–Whitney *U*; ^c^ Fisher’s exact test; ^d^ Log-rank test

^e^In patients with haemodynamic instability

### Secondary outcomes

#### Intraoperative arrhythmias

IG patients developed fewer arrhythmias than CG patients (2.2% vs 0.7%; *p*=0.172).

#### Postoperative complications

Postoperative complications are shown in Table 3. Postoperative complications rates were 42.3% in the IG versus 45.2% in CG (*p*=0.489). Patients in the IG had fewer cardiovascular complications (18.8% vs 7.2%; *p*< 0.001), fewer respiratory complications (15.1% vs 3.6%; *p*<0.001) and postoperative infections (21% vs 3.9%; *p*<0.001). However, IG patients had more renal complications (12.1% vs 33.7%; *p*<0.001). No differences in postoperative renal complications were observed between groups in patients with normal creatinine value at hospital admission. (Additional file 4).

#### Fluid volumes, vasopressor doses and perioperative transfusion

Details of fluid volumes and vasopressor doses in both groups are shown in Table 4.

**Table 4:** Total fluid volumes, vasopressor doses and perioperative blood transfusion

	Control (CG) ***n***=272		Intervention (IG) ***n***=279		***p***-value
	*n* (%)	Median (IQR)	*n* (%)	Median (IQR)	
**Fluid volumes**					
**Total fluids (ml)**	272 (100%)	2600 (IQR 1700 to 2700)	279 (100%)	850 (IQR 750 to 1050)	0.001
Fluid creep -antibiotic prophylaxis- (ml)	272 (100%)	200 (IQR 200 to 200)	279 (100%)	200 (IQR 100 to 200)	0.001
Intraoperative fluids (ml)	272 (100%)	2500 (IQR 2000 to 2500)	279 (100%)	700 (IQR 550 to 900)	0.001
**Crystalloids (ml)**	253 (94.5%)	2000 (IQR 2000 to 2000)	278 (99.6%)	650 (IQR 550 to 850)	0.001
Saline (ml)	68 (25.0%)	1000 (IQR 1000 to 1000)	267 (95.7%)	650 (IQR 550 to 850)	0.001
Lactated Ringer (ml)	201 (73.9%)	1000 (IQR 1000 to 1000)	12 (4.3%)	1050 (IQR 850 to 1237)	0.889
Isofundin® (ml)	22 (8.1%)	500 (IQR 500 to 500)	-	-	-
**Colloids (ml)**	161 (59.2%)	500 (IQR 500 to 500)	25 (9.0%)	300 (IQR 200 to 500)	0.001
Voluven® (ml)	153 (56.3%)	500 (IQR 500 to 500)	25 (9.0%)	300 (IQR 200 to 500)	0.001
Gelaspan® (ml)	11 (4.0%)	500 (IQR 500 to500)	-	-	-
**Vasopressor**	108 (39.7%)		71 (25.5%)		<0.001
Ephedrine (mg)	108 (39.7%)	15 (IQR 10 to 30)	65 (23.3%)	10 (IQR 10 to 20)	0.002
Phenylephrine (mg)	-	-	11 (4.0%)	100 (IQR 50 to 150)	-
Noradrenaline (mg)	2 (0.7%)	3.5 (IQR 2 to 3.5)	-	-	-
**Blood transfusion**	200 (73.5%)	-	124 (44.4%)	-	<0.001
Number of PRBC^a^		2 (IQR 2 to 4)		2 (IQR 1 to 2)	<0.001
**Fluid Challenge Responder**	-	-	82 (29.4%)		-

Median (IQR 25th percentile to 75th percentile)

*PRBC* packed red blood cells

^a^About transfused patients

29.4% of IG patients were responders to the first fluid bolus performed. Patients in the IG received less fluid [2.600 ml (IQR 1700 to 2700) vs 850 ml (IQR 750 to 1050); *p*=0.001] and vasopressors (39.7% vs 25.5%; *p*<0.001) than CG. Lactated Ringer’s was the fluid most used during the intraoperative period in CG patients (73.9% vs 4.3%; *p*<0.001) while saline was chosen more often in the intraoperative period in IG (25% vs 95.7%; *p*=0.001). Fewer patients in IG received colloids than in CG (59.2% vs 9%; *p*<0.001).

Fewer patients required packed red blood cells (PRBC) transfusion in IG (73.5% vs 44.4%; *p*<0.001), with a lower median number of PRBC among transfused patients in IG [2 (IQR 2 to 4) vs 2 (IQR 1 to 2); *p*<0.001].

#### Length of stay and survival within 12 months of surgery

The median length of stay was shorter for patients in the IG (median days: 11 vs 8; *p* < 0.001) (Table 3).

**Fig. 3:**
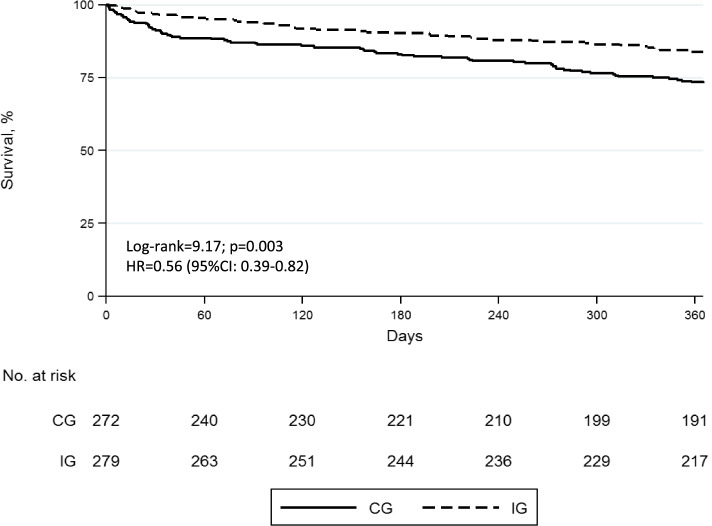
Kaplan–Meier survival curves according to group allocation. Crude hazard ratio for 1-year survival

Demographic and clinical variables associated with 1-year mortality in the bivariate analysis appear in Additional file 2. Figure 3 shows the Kaplan–Meier survival curves for both groups. The likelihood of 1-year survival was higher in IG (log-rank test=9.17; *p* = 0.003) (see Fig. 3), with a crude HR of 0.56 (95% CI 0.39 to 0.82). Multivariate analysis (Additional file 3) showed that independent prognostic factors for 1-year survival were as follows: age (HR 1.09; 95% CI 1.05 to 1.12), male gender (HR 2.10; 95% CI 1.43 to 3.11), low (HR 2.33; 95% CI 1.29 to 4.23) and high comorbidity (HR 2.84; 95% CI 1.67 to 4.83), according to the Charlson Index, postoperative cardiovascular complications (HR 3.85; 95% CI 2.49 to 5.96), need for reintervention (HR 5.31; 95% CI 1.58 to 17.86) and belonging to the intervention group*. The adjusted HR for the IG was 0.61 (95% CI 0.39 to 0.95).

## Discussion

The use of GDHT guided by non-invasive haemodynamic monitoring in patients undergoing hip fracture surgery within an ERP, was associated with a reduction in intraoperative complications (haemodynamic instability, arrhythmias) and postoperative cardiovascular, respiratory and infectious complications, but not postoperative renal complications. This strategy was also associated with a shorter hospital stay and increased survival 1 year after surgery.

Preoperative chronic conditions and insufficient preoperative optimization in this patient profile, associated with intraoperative conditions such as bleeding and hypovolemia, predispose these elderly patients to haemodynamic instability or arrhythmias during surgery (Alecu et al. 2010); (Rocos et al. 2017). The occurrence of intraoperative complications may compromise the balance between tissue oxygen delivery and oxygen consumption and increase the patient’s susceptibility to postoperative complications (Merry and Mitchell 2018); (Beecham et al. 2020).

Our results showed a significant decrease in intraoperative haemodynamic instability episodes in patients in the IG, similarly to a recently published study (Davies et al. 2019). In addition, patients in the IG had fewer postoperative cardiovascular, respiratory and infectious complications. These results may be due to improvements in haemodynamic control of these patients as a result of GDHT guided by a non-invasive monitoring system implemented. One of its main objectives is to avoid intraoperative hypoperfusion (Brienza et al. 2019) which may have been reflected in fewer postoperative complications in IG.

A higher incidence of postoperative acute kidney injury (AKI) found in IG may be due to several reasons. First, patients in the IG have more risk factors for postoperative AKI, including age, female sex, hypertension and chronic kidney disease. (Meersch et al. 2017). Secondly, patients treated with GDHT received higher amounts of 0.9% saline. Hyperchloremic acidosis associated with saline infusion is detrimental to renal artery blood flow velocity and renal cortical tissue perfusion (Chowdhury et al. 2012). Third, the AKI definition itself. A single and transient, postoperative serum creatinine elevation above a very sensitive level was considered a renal complication. An isolated elevation could neither be associated with kidney cell damage (Hahn 2015) nor be significant compared to baseline value. In patients with normal creatinine value at hospital admission, no differences in the risk of suffering postoperative renal complications were observed between the groups. The limitations of AKI definition and the influence on mortality only by postoperative creatinine elevations associated with kidney cell damage, may explain a significant decrease in mortality 1 year after surgery, despite an increase in postoperative renal complications in IG.

The GDHT protocol applied in this study differs from that used in the most recent trials in this patient profile (Bartha et al. 2013; Davies et al. 2019; Moppett et al. 2015). The GDHT protocol used by Bartha and colleagues includes fluid resuscitation prior to anaesthetic induction, intraoperative use of vasoactive support if SAP declined by more than 30% from pre-anaesthesia values, and optimisation with fluids and dobutamine for stroke volume (SV) and DO_2_ respectively. Colloid therapy to optimize SV was protocolised by Moppett and co-workers in their IG. Finally, Davies and colleagues applied a GDHT protocol based on SV optimisation by crystalloids and mean arterial pressure maintenance above 30% of baseline values with vasopressors.

Our IG shows a similar percentage of responders to the first fluid challenge as previously described (Bartha et al. 2013). This lower-than-expected percentage can be explained by the optimisation starting immediately after admission to hospital, according to the ERP applied in both patient cohorts. Total fluid used in our IG is comparable or slightly less than the amounts given in two previous studies’ intervention groups: 1.078 (Bartha et al. 2013) and 850 ml (Davies et al. 2019) respectively and slightly higher than the volume given in another previous intervention group: 750 ml (Moppett et al. 2015). A lower use of vasopressors in our IG stands out, probably because of following a decision algorithm. The non-application of a haemodynamic algorithm may lead to an early use of vasopressors, even if it is not physiologically appropriate for the patient’s condition. No IG patients were treated intraoperatively with dobutamine, probably due to the establishment of an individualized CI goal and by the absence of intraoperative pathophysiological tributary situations. Fewer patients had been transfused in our IG. In addition to the use of less bleeding surgical techniques (Yu et al. 2015), patients in IG may suffered less haemodilution than patients in CG due to lower amount of fluids received (Ince 2015).

We found a reduction in hospital stay and a significant increased survival in IG patients throughout the first year after surgery. These findings can be explained by the significant reduction in intraoperative complications and postoperative cardiovascular, respiratory and infectious complications, and surgical reinterventions (Monk et al. 2015); (Roche et al. 2005). After adjusting for potential confounding variables, IG membership was a protective factor for 1-year mortality.

The results of this study suggest that not only haemodynamic strategy we perform on our patients will influence their outcomes, the type of fluid used during major surgery may also affect postoperative results (Heming et al. 2020). The results of the fluid infusion strategy used cannot be evaluated without considering the type of drug used.

Our study has some limitations. It was a single-centre, non-randomized design with a 3-year gap between the two study groups. However, no changes were made between groups in the ERP, nor in the composition of the multidisciplinary team or in the care protocols during the study period. IG recruitment rate was lower than expected, probably because of the real emergency surgery status applied to hip fracture patients in this hospital since 2010. Patients with contraindications for haemodynamic monitoring, with poor-quality signal obtained or with preoperative psychomotor agitation that prevented haemodynamic monitoring were excluded only from the IG. However, our exclusion rate for these reasons (9%) is lower than previously reported (Davies et al. 2019), but we cannot rule out that these excluded patients had worse clinical status on arrival in the operating theatre.

Uncontrolled before-and-after study provides less quality evidence than randomized controlled trials (RCT) (Sedgwick 2014). However, this design offers valuable insights into the potential benefits of GDHT protocols under real-life conditions and can complement evidence from RCT (Saugel et al. 2019). Moreover, this is the largest sample size published to date evaluating the effect of GDHT guided by non-invasive haemodynamic monitoring during hip fracture surgery.

## Conclusions

In patients undergoing hip fracture surgery, the use of a GDHT protocol guided by non-invasive haemodynamic monitoring was associated with a reduction in intraoperative complications and postoperative cardiovascular, respiratory and infectious, but not postoperative renal complications. This strategy was also associated with shorter hospital stay and higher survival 1 year after surgery.

## Supplementary Information

**Additional file 1..** Intraoperative cardiac index goal groups.

**Additional file 2..** Bivariant analysis. Prognostic factors of 1-year mortality. Crude Hazard Ratio (HR) and statistical significance according to bivariate COX regression models.

**Additional file 3..** Multivariant analysis. Independent prognostic factors for mortality. Adjusted HR for 1-year survival.

**Additional file 4..** Renal complications according creatinine at admission and group allocation.

## Data Availability

The datasets used and/or analysed during this study are available from the corresponding author on reasonable request

## Abbreviations

AKI: Acute kidney injury
CG: Control group
CI: Cardiac Index
DO_2_: Oxygen delivery
ERP: Enhanced recovery pathway
FB: Fluid bolus
GDHT: Goal-directed haemodynamic therapy
HR: Hazard ratio
IG: Intervention group
IQR: Interquartile range
MDRD: Modification of Diet in Renal Disease
METS: Metabolic equivalents
PACU: Post-anaesthetic care unit
PRBC: Packed red blood cells
SAP: Systolic arterial pressure
SpO_2_: Oxygen saturation
SV: Stroke volume
SVI: Indexed stroke volume
VO_2_: Oxygen consumption
