# Supplementary material for: Effect of goal-directed haemodynamic therapy guided by non-invasive monitoring on perioperative complications in elderly hip fracture patients within an enhanced recovery pathway
Source: Perioper Med (Lond). 2022 Aug 10;11:46. doi: 10.1186/s13741-022-00277-w (PMC9364538; doi:10.1186/s13741-022-00277-w)
Supplement: Supplementary file 1 — Additional file 1.. Intraoperative cardiac index goal groups. [file 13741_2022_277_MOESM1_ESM.docx]

**Additional file 1. Intraoperative cardiac index goal groups:**

|  | **Age (years)** | 65-74 | 75-85 | > 85 |
| --- | --- | --- | --- | --- |
| **Functional Capacity (METS)** |  |  |  |  |
| < 4 METS |  | 2.4 | 2.2 | 2.2 |
| Between 4-6 METS |  | 2.6 | 2.4 | 2.2 |
| > 6 METS |  | 2.6 | 2.4 | 2.2 |

*Intraoperative cardiac index goal groups according to age and METS*

*Cardiac index expressed in L/min/m2*
