# Supplementary material for: Effect of goal-directed haemodynamic therapy guided by non-invasive monitoring on perioperative complications in elderly hip fracture patients within an enhanced recovery pathway
Source: Perioper Med (Lond). 2022 Aug 10;11:46. doi: 10.1186/s13741-022-00277-w (PMC9364538; doi:10.1186/s13741-022-00277-w)
Supplement: Supplementary file 2 — Additional file 2.. Bivariant analysis. Prognostic factors of 1-year mortality. Crude Hazard Ratio (HR) and statistical significance according to bivariate COX regression models. [file 13741_2022_277_MOESM2_ESM.docx]

**Additional file 2. Bivariant analysis. Prognostic factors of one-year mortality. Raw hazard ratio (HR) and statistical significance according to bivariate COX regression models.**

|  | **No death**  **n=436** | **Death**  **n=115** | **p-value** | **crude HR** | **CI 95%** |
| --- | --- | --- | --- | --- | --- |
| **Age** |  |  | <0.001^a^ |  |  |
| 65 to < 75 years | 44 (97.8%) | 1 (2.2%) |  | 1 |  |
| 75 to < 85 years | 189 (85.1%) | 33 (14.9%) |  | 7.25 | 0.99 to 53.04 |
| ≥85 years | 203 (71.5%) | 81 (28.5%) |  | 14.85 | 2.07 to 106.7 |
|  | 84.2 ± 6.8 | 88.1 ± 6.0 | <0.001^b^ | 1.09 | 1.06 to 1.12 |
| **Gender** |  |  | <0.001^a^ |  |  |
| Female | 332 (83.2%) | 67 (16.8%) |  | 1 |  |
| Male | 104 (68.4%) | 48 (31.6%) |  | 2.11 | 1.46 to 3.06 |
| **ASA** |  |  | <0.001^a^ |  |  |
| I – II | 118 (93.7%) | 8 (6.3%) |  | 1 |  |
| III-IV | 318 (74.8%) | 107 (25.2%) |  | 4.47 | 2.18 to 9.17 |
| **Charlson comorbidity index** |  |  | <0.001^a^ |  |  |
| Absence of comorbidity (0-1) | 212 (90.6%) | 22 (9.4%) |  | 1 |  |
| Low comorbidity (2) | 81 (75.7%) | 26 (24.3%) |  | 2.86 | 1.62 to 5.04 |
| High comorbidity (3 or more) | 143 (68.1%) | 67 (31.9%) |  | 3.87 | 2.39 to 6.26 |
| **Total number of drugs** |  |  | <0.006^a^ |  |  |
| ≤ 4 drugs | 136 (86.6%) | 21 (13.4%) |  | 1 |  |
| > 4 drugs | 300 (76.1%) | 94 (23.9%) |  | 1.84 | 1.15 to 2.96 |
| **Antiplatelet agents** |  |  | 0.607^a^ |  |  |
| No | 272 (79.8%) | 69 (20.2%) |  | 1 |  |
| AAS100mg | 99 (76.2%) | 31 (23.8%) |  | 1.19 | 0.78 to 1.82 |
| AAS>100mg/Clopidogrel | 65 (81.3%) | 15 (18.8%) |  | 0.90 | 0.52 to 1.58 |
| **Anticoagulants** |  |  | 0.095^a^ |  |  |
| No | 404 (80.0%) | 101 (20.0%) |  | 1 |  |
| Yes | 32 (69.6%) | 14 (30.4%) |  | 1.63 | 0.93 to 2.85 |
| **Type of fracture** |  |  | 0.811^a^ |  |  |
| Intra-articular | 195 (79.6%) | 50 (20.4%) |  |  |  |
| Extra-articular | 241 (78.8%) | 65 (21.2%) |  | 1.06 | 0.73 to 1.53 |
| **Haemoglobin at admission** |  |  | <0.001^a^ |  |  |
| Hb>12 g/dl | 276 (84.1%) | 52 (15.9%) |  | 1 |  |
| Hb ≤12 g/dl | 160 (71.7%) | 63 (28.3%) |  | 1.93 | 1.34 to 2.79 |
| **Surgical delay** |  |  | 0.693^a^ |  |  |
| 0 - 48 hours | 268 (78.6%) | 73 (21.4%) |  | 1 |  |
| > 48 hours | 168 (80.0%) | 42 (20.0%) |  | 0.92 | 0.63 to 1.35 |
| **Anaesthesia** |  |  | 0.914^a^ |  |  |
| Spinal | 392 (79.2%) | 103 (20.8%) |  | 1 |  |
| General | 44 (78.6%) | 12 (21.4%) |  | 1.05 | 0.58 to 1.91 |
| **Surgical technique** |  |  | 0.074^a^ |  |  |
| Hip prosthesis | 165 (80.5%) | 40 (19.5%) |  | 1 |  |
| Dynamic hip screw | 134 (74.9%) | 45 (25.1%) |  | 1.34 | 0.88 to 2.06 |
| Intramedullary nail | 134 (83.2%) | 27 (16.8%) |  | 0.87 | 0.54 to 1.42 |
| Others | 3 (50.0%) | 3 (50.0%) |  | 2.94 | 0.91 to 9.52 |
| **Blood transfusion** |  |  | 0.008^a^ |  |  |
| No | 192 (84.6%) | 35 (15.4%) |  | 1 |  |
| Yes | 244 (75.3%) | 80 (24.7%) |  | 1.71 | 1.15 to 2.55 |
| **Surgery time (minutes)** | 85 (IQR 65 to 115) | 80 (IQR 60 to 111) | 0.357^b^ | 1.00 | 0.99 to 1.00 |
| **Intraoperative complications** |  |  |  |  |  |
| **Haemodynamic instability** |  |  | 0.011^a^ |  |  |
| No | 305 (82.2%) | 66 (17.8%) |  | 1 |  |
| Yes | 131 (72.8%) | 49 (27.2%) |  | 1.67 | 1.15 to 2.41 |
| **Arrhythmias** |  |  | 0.675^c^ |  |  |
| No | 430 (79.2%) | 113 (20.8%) |  | 1 |  |
| Yes | 6 (75.0%) | 2 (25.0%) |  | 1.45 | 0.36 to 5.86 |
| **Postoperative complications** |  |  |  |  |  |
| **Cardiovascular** |  |  | <0.001^a^ |  |  |
| No | 403 (84.0%) | 77 (16.0%) |  | 1 |  |
| Yes | 33 (46.5%) | 38 (53.5%) |  | 4.79 | 3.24 to 7.07 |
| **Major** |  |  | <0.001^c^ |  |  |
| No | 429 (81.3%) | 99 (18.8%) |  | 1 |  |
| Yes | 7 (30.4%) | 16 (69.6%) |  | 6.68 | 3.93 to 11.34 |
| **Minor** |  |  | <0.001^a^ |  |  |
| No | 408 (81.8%) | 91 (18.2%) |  | 1 |  |
| Yes | 28 (53.8%) | 24 (46.2%) |  | 3.24 | 2.07 to 5.09 |
| **Respiratory** |  |  | <0.001^a^ |  |  |
| No | 408 (81.6%) | 92 (18.4%) |  | 1 |  |
| Yes | 28 (54.9%) | 23 (45.1%) |  | 3.07 | 1.95 to 4.86 |
| **Haematological** |  |  | 0.604 ^a^ |  |  |
| No | 416 (78.9%) | 111 (21.1%) |  | 1 |  |
| Yes | 20 (83.3%) | 4 (16.7%) |  | 0.80 | 0.29 to 2.16 |
| **Renal** |  |  | <0.001^a^ |  |  |
| No | 352 (83.0%) | 72 (17.0%) |  | 1 |  |
| Yes | 84 (66.1%) | 43 (33.9%) |  | 2.35 | 1.61 to 3.43 |
| **Infections** |  |  | 0.064^a^ |  |  |
| No | 388 (80.3%) | 95 (19.7%) |  | 1 |  |
| Yes | 48 (70.6%) | 20 (29.4%) |  | 1.61 | 0.99 to 2.61 |
| **Surgical reintervention** |  |  | 0.109^c^ |  |  |
| No | 433 (79.4%) | 112 (20.6%) |  | 1 |  |
| Yes | 3 (50.0%) | 3 (50.0%) |  | 3.26 | 1.04 to 10.28 |
| **Length of stay (days)** | 9 (IQR 7 to 13) | 10 (IQR 8 to 17) | 0.008^b^ | 1.02 | 1.00 to 1.03 |
| **Destination after discharge** |  |  | <0.007^a^ |  |  |
| Family home | 132 (86.8%) | 20 (13.2%) |  | 1 |  |
| Convalescence | 226 (83.7%) | 44 (16.3%) |  | 1.24 | 0.73 to 2.11 |
| Residence | 78 (72.2%) | 30 (27.8%) |  | 2.24 | 1.27 to 3.94 |
| **Thirty-day readmission** |  |  | 0.006^a^ |  |  |
| No | 402 (83.8%) | 78 (16.3%) |  | 1 |  |
| Yes | 34 (68.0%) | 16 (32.0%) |  | 2.21 | 1.29 to 3.79 |
| **Group allocation** |  |  | 0.003^a^ |  |  |
| Control group | 201 (73.9%) | 71 (26.1%) |  | 1 |  |
| Intervention group | 235 (84.2%) | 44 (15.8%) |  | 0.56 | 0.39 to 0.82 |

n (%); median (IQR 25^th^ percentile to 75^th^ percentile).

^a^ Pearson χ^2^; ^b^ Mann–Whitney U; ^c^ Fisher’s exact test.
