# Supplementary material for: Effect of goal-directed haemodynamic therapy guided by non-invasive monitoring on perioperative complications in elderly hip fracture patients within an enhanced recovery pathway
Source: Perioper Med (Lond). 2022 Aug 10;11:46. doi: 10.1186/s13741-022-00277-w (PMC9364538; doi:10.1186/s13741-022-00277-w)
Supplement: Supplementary file 3 — Additional file 3.. Multivariant analysis. Independent prognostic factors for mortality. Adjusted HR for 1-year survival. [file 13741_2022_277_MOESM3_ESM.docx]

**Additional file 3. Multivariant analysis. Independent prognostic factors for mortality. Adjusted HR for one-year survival.**

|  | **Adjusted HR** | **95% CI** | **p-value** |
| --- | --- | --- | --- |
| **Age** | 1.09 | 1.05 to 1.12 | <0.001 |
| **Gender** |  |  |  |
| Female | 1 |  |  |
| Male | 2.10 | 1.43 to 3.11 | <0.001 |
| **ASA** |  |  |  |
| I-II | 1 |  |  |
| III-IV | 1.92 | 0.87 to 4.24 | 0.105 |
| **Charlson Index** |  |  |  |
| Absence of comorbidity (0-1) | 1 |  |  |
| Low comorbidity (2) | 2.33 | 1.29 to 4.23 | 0.005 |
| High comorbidity (3 or more) | 2.84 | 1.67 to 4.83 | <0.001 |
| **Intraoperative haemodynamic instability** |  |  |  |
| No | 1 |  |  |
| Yes | 1.44 | 0.97 to 2.14 | 0.073 |
| **Postoperative cardiovascular complications** |  |  |  |
| No | 1 |  |  |
| Yes | 3.85 | 2.49 to 5.96 | <0.001 |
| **Postoperative renal complications** |  |  |  |
| No | 1 |  |  |
| Yes | 1.47 | 0.96 to 2.25 | 0.075 |
| **Reintervention** |  |  |  |
| No | 1 |  |  |
| Yes | 5.31 | 1.58 to 17.86 | 0.007 |
| **Group allocation** |  |  |  |
| Control group (CG) | 1 |  |  |
| Intervention group (IG) | 0.61 | 0.39 to 0.95 | 0.029 |
