# Supplementary material for: Effect of goal-directed haemodynamic therapy guided by non-invasive monitoring on perioperative complications in elderly hip fracture patients within an enhanced recovery pathway
Source: Perioper Med (Lond). 2022 Aug 10;11:46. doi: 10.1186/s13741-022-00277-w (PMC9364538; doi:10.1186/s13741-022-00277-w)
Supplement: Supplementary file 4 — Additional file 4.. Renal complications according creatinine at admission and group allocation. [file 13741_2022_277_MOESM4_ESM.docx]

Additional file 4. Renal complications according to creatinine at admission and group allocation.

|  | Control (CG) | Intervention (IG) | p-value |
| --- | --- | --- | --- |
| Creatinine at admission ≤ 1.09 mg/dl | n=178 | n=175 |  |
| Renal Postoperative complications | 11 (6.2%) | 21 (12.0%) | 0.057^a^ |
| Creatinine at admission > 1.09 mg/dl | n=93 | n=104 |  |
| Renal Postoperative complications | 22 (23.7%) | 73 (70.2%) | <0.001^a^ |

N (%); ^a^ χ^2^ de Pearson
